# Femoropopliteal arterial bone formation

**DOI:** 10.1093/ehjcr/ytaf626

**Published:** 2025-11-29

**Authors:** Eiji Karashima, Sho Torii, Yuki Matsumoto

**Affiliations:** Department of Cardiology, Shimonoseki City Hospital, 1-13-1 Kouyou-chou, Shimonoseki, Yamaguchi 750-8520, Japan; Department of Cardiology, Tokai University School of Medicine, 143 Shimokasuya, Isehara, Kanagawa 259-1193, Japan; Department of Cardiology, Tokai University School of Medicine, 143 Shimokasuya, Isehara, Kanagawa 259-1193, Japan

## Case description

A 77-year-old female with diabetes and end-stage renal disease (ESRD) on dialysis developed a right toe ulcer with severe lower leg infection, classified as Rutherford Category 6. Angiography of the right leg revealed femoropopliteal artery stenosis and posterior tibial artery occlusion. Intravascular ultrasound (IVUS) revealed a 360° calcium arc, a minimal lumen area of 3.4 mm^2^, and a reference vessel diameter of 4.6 mm.

**Figure 1:**
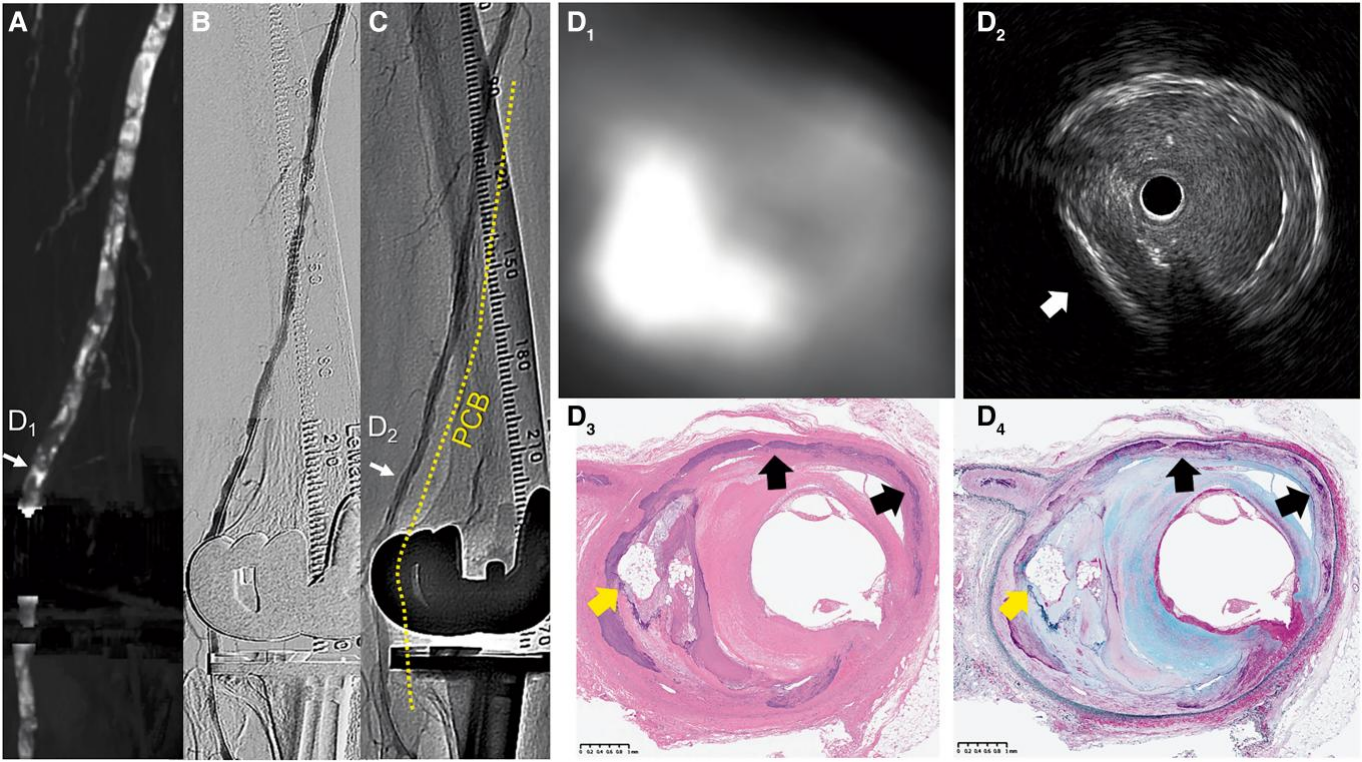
(*A*) Baseline computed tomography angiography. (*B* and *C*) Angiography before and after endovascular treatment. (*D_1_–D_4_*) Multimodality images at the same arterial level. (*D_1_*) Computed tomography image showing focal thick arterial calcification with high Hounsfield units (722 HU) comparable to bone (300–3000 HU). (*D_2_*) Intravascular ultrasound revealing deep calcification with acoustic shadowing (arrow). (*D_3_* and *D_4_*) Histological sections of the femoropopliteal artery showing intimal intramembranous ossification with bone marrow elements. (*D_3_*) Haematoxylin and eosin stain. (*D_4_*) Movat pentachrome stain. Scale bars are indicated on the panels. Arrows indicate intimal bone formation within the thick calcified arterial wall and sheet-like medial calcification. CT, computed tomography; PCB, paclitaxel-coated balloon.

She underwent endovascular treatment with a 4.0 × 150 mm scoring balloon, followed by a 4.0 × 200 mm paclitaxel-coated balloon (PCB). Due to persistent wound infection, she required below-the-knee amputation, followed by above-the-knee amputation 57 days later despite wound care and antibiotics. Multimodal imaging at the same arterial level revealed extensive arterial calcification (*Figure 1D1–D4*). Computed tomography (CT) showed focal thick arterial calcification with high Hounsfield units, which was similar to that of bone of thigh (*Figure 1D1*). Intravascular ultrasound confirmed deep calcification with acoustic shadow (*Figure 1D2*).^1^ Unlike other areas, the region with deep calcification and bone formation showed limited expansion despite scoring-balloon predilation. Pathological examination revealed bone formation within the severely calcified arterial wall (*Figure 1D3* and *D4*).

In ESRD, disordered mineral metabolism and chronic inflammation promote osteogenic transdifferentiation of vascular smooth muscle cells, predisposing to medial calcification and osseous metaplasia. These processes may occur via intramembranous ossification and likely contributed to the extreme vessel non-compliance observed in this case.^2^ Calcium-modifying technologies may be considered for refractory, non-compliant lesions when severe calcification or osseous metaplasia is suspected. Previous pathological study demonstrated that the prevalence of bone formation is much higher than that of coronary arteries^3,4^; however, the clinical impact of bone formation in femoropopliteal arteries was not well evaluated before. To our knowledge, this is the first report demonstrating bone formation in femoropopliteal artery calcification using multimodality imaging validated by histology.

**Consent:** The author/s confirm that written consent for submission and publication of this case report including image(s) and associated text has been obtained from the patient in line with the COPE guidelines.

**Funding:** None declared.

**Data availability:** The data underlying this article are available in the article and in its online supplementary material.

## References

[1] Fujihara M, Kurata N, Yazu Y, Mori S, Tomoi Y, Horie K, et al Clinical expert consensus document on standards for lower extremity artery disease of imaging modality from the Japan Endovascular Treatment Conference. Cardiovasc Interv Ther 2022;37:597–612. 35852760 10.1007/s12928-022-00875-x

[2] Mori H, Torii S, Kutyna M, Sakamoto A, Finn AV, Virmani R. Coronary artery calcification and its progression: what does it really mean? JACC Cardiovasc Imaging 2018;11:127–142. 29301708 10.1016/j.jcmg.2017.10.012

[3] Torii S, Mustapha JA, Narula J, Mori H, Saab F, Jinnouchi H, et al Histopathologic characterization of peripheral arteries in subjects with abundant risk factors: correlating imaging with pathology. JACC Cardiovasc Imaging 2019;12:1501–1513. 30553660 10.1016/j.jcmg.2018.08.039

[4] Kato T, Torii S, Nakamura N, Aihara K, Terabe Y, Iida O, et al Pathological analysis of medial and intimal calcification in lower extremity artery disease: impact of hemodialysis. JACC Adv 2023;2:100656. 38938733 10.1016/j.jacadv.2023.100656 PMC11198496

